# Accessibility in proteins and RNAs interactions prediction with machine learning: are we overlooking non-experts?

**DOI:** 10.1093/bib/bbag226

**Published:** 2026-05-11

**Authors:** Bruno R Florentino, Robson P Bonidia, André C P L F de Carvalho

**Affiliations:** Institute of Mathematical and Computer Sciences, University of São Paulo, Avenida Trabalhador São-Carlense 400, Centro, São Carlos, SP 13560-924, Brazil; Global South Artificial Intelligence for Pandemic and Epidemic Preparedness & Response Network (AI4PEP); Institute of Mathematical and Computer Sciences, University of São Paulo, Avenida Trabalhador São-Carlense 400, Centro, São Carlos, SP 13560-924, Brazil; Global South Artificial Intelligence for Pandemic and Epidemic Preparedness & Response Network (AI4PEP); Department of Computer Science, Federal University of Technology-Paraná (UTFPR), Cornélio Procópio 80230-901, Brazil; Institute of Mathematical and Computer Sciences, University of São Paulo, Avenida Trabalhador São-Carlense 400, Centro, São Carlos, SP 13560-924, Brazil; Global South Artificial Intelligence for Pandemic and Epidemic Preparedness & Response Network (AI4PEP)

**Keywords:** protein interactions, RNA interactions, machine learning, end-to-end, accessibility

## Abstract

The increasing growth in the volume of biomolecular data has introduced significant challenges for extracting meaningful molecular-level insights, particularly in predicting interactions between biological sequences such as DNA, RNA, and proteins. These interactions are fundamental to complex biological processes, including gene regulation and immune response. Artificial Intelligence (AI) has played a major role in advancing discoveries in this field, enabling the identification of novel interactions, as demonstrated by various predictive modeling studies. Despite the growing number of scientific publications in this domain, accessibility to practical computational tools has not progressed at the same pace. Existing studies differ substantially in availability: some provide only methodological descriptions, others release source code exclusively for experimental reproducibility, and only a limited number deliver fully automated solutions ready for broad use. Given this context, this paper investigates state-of-the-art studies in biological sequence interaction prediction, emphasizing the public accessibility and usability of available tools, especially for researchers who are not experts in AI or computational methods. We compile and discuss the input requirements of current tools, along with the types of outputs they generate, enabling users to better understand the scenarios in which each solution can be effectively applied. Furthermore, we analyze accessibility-related aspects to support informed selection of tools according to user expertise, ranging from web-based servers with pretrained models that require minimal computational skills to fully end-to-end frameworks capable of training new models on user-defined datasets, though often lacking user-friendly interfaces.

## Introduction

Advances in modern genetic sequencing technologies have significantly expanded the generation of biological sequences, such as proteins and RNAs, with known primary structure [1, 2]. Consequently, a wide range of species information is now cataloged within these repositories [3–5]. This scenario highlights the need for computational techniques capable of handling such large volumes of data and leveraging them to drive discoveries [5, 6]. One area that has benefited from computational techniques, particularly machine learning (ML), is the prediction of interactions between biological sequences. Laboratory-based experimental methods are often expensive and time-consuming to perform [7–11]. Considering this, there is a growing use of computational approaches to predict these interactions and accelerate the experimental phase [8, 11].

As an example, discovering and mapping protein–protein interactions (PPIs) is valuable [12], as they are associated with various types of diseases, such as cancer [13–16], infectious diseases [14, 17], and neurodegenerative disorders [14, 16]. In parallel, RNAs are also essential molecules, as they play a crucial role in gene expression regulation [7] and chromatin state through their interactions, influencing not only the regions near their transcription sites but also the more distant genomic regions [18].

Nevertheless, despite the availability of numerous ML libraries and platforms for all users, not everyone has the necessary skills to initiate projects using this technology [7, 19]. This skill gap can prevent many researchers and professionals from taking advantage of computational tools that rely directly on programming for their implementation. To deal with this limitation, the state-of-the-art shows tools that are capable of training models and predicting new pairs of interactions [8–10, 13, 20].

These approaches, when fitting a model to discover novel interaction pairs, require a set of known interactions to be used for training and testing the predictive model [10, 12]. Furthermore, these models require complementary input to characterize each biological sequence, with a common technique being the use of the primary structure [8, 12]. For example, consider A1L167, a human enzyme, a special case of the primary structure of a protein. In this sequence, represented by the letters MKELQDIARLSD..., each letter corresponds to a specific amino acid. This sequence illustrates the linear arrangement of amino acids that form the protein backbone, which is characterized as categorical and unstructured data [21]. Subsequently, these tools process these inputs either automatically or manually, by training an ML model to predict new pairs based on the context of the known interactions used during training [20, 22].

These approaches for interaction prediction can vary significantly. Previous systematic studies have already explored the diversity of computational techniques applied in this field [11, 78–83]. However, when it comes to aspects related to the accessibility of these tools for non-expert users, no systematic investigations were identified that evaluate and classify them. Analyzing this factor is essential, as understanding the degree of accessibility enables researchers without extensive training in AI to select approaches that are most compatible with their skill level, available resources, and specific requirements.

### Objectives

Given this context, the main goal of this mapping is to understand and identify validated state-of-the-art tools that can be used by non-experts in ML to predict new interactions between biological sequences, including PPIs, RNA–protein interactions (RPIs), and RNA–RNA interactions (RRIs). For such, it is essential to comprehend all aspects of these tools, including their availability, accessibility for non-experts in ML, input file types, modeling techniques, outputs, and validation processes.

Furthermore, this mapping includes only tools whose source code is made available by the authors or those hosted on a server or other form of public access. This ensures that new users do not need to re-implement a described methodology to use it for training and predicting new interactions. Additionally, only validated tools will be considered, those whose methodology has been tested with more than one dataset and whose performance has been compared with at least one other tool, providing minimal validation before being applied to new datasets by users.

### Research questions

This study is guided by the following research questions (RQs):

**RQ1:** Regarding tools capable of predicting interactions between biological molecules, including PPIs, RPIs, and RRIs, what type of input data do the most modern predictive models require, and what limitations are imposed by these data requirements?

**RQ2:** Considering that prediction results may be used in different biological analysis workflows, what type of outputs do current prediction tools provide, and how useful or informative are these outputs for downstream interpretation?

**RQ3**: Since web-based prediction servers allow users to perform interaction predictions without the need for configuration or local execution, what online servers currently provide pretrained models for predicting novel interactions, and which organisms and interaction types do they support?

**RQ4:** Given the need to expand the applicability and accessibility of these technologies, do existing tools offer end-to-end automated pipelines for model training, and what level of user expertise is required to configure and deploy such models?

Therefore, this review maps key aspects of current interaction prediction tools, both to inform potential users and to highlight gaps for ML researchers. Specifically, we aim to understand what is required to effectively use these tools, including the type and nature of input data they demand (RQ1), thereby defining the practical scenarios in which they can be applied. Additionally, we investigate the types of outputs produced (RQ2), beyond the predicted interactions themselves, identifying whether complementary or explainable results are provided. We also assess the degree of automation offered by these solutions, examining how tools with pretrained models operate (RQ3), as well as the workflow and capabilities of those that allow users to train new predictive models (RQ4). This study seeks to build a comprehensive understanding of these tools not by re-examining the modeling techniques already well covered in prior secondary studies, but by focusing on their usability, availability, required inputs, and delivered outputs, aspects that remain underexplored yet crucial for real-world adoption.

### The main contributions from this mapping

Our main contribution is to focus on the identification and understanding of the existing published tools from the perspective of real-world applications, both for non-specialists in coding and for AI researchers. For such, this study is structured around three main pillars: model inputs, outputs, and the level of automation. These are important practical aspects, as the type of input often determines the kind of molecular information that the user must provide to predict target interactions. Additionally, the output type defines what the model is able to deliver to the user, beyond the predicted interactions. The level of automation reflects how easily the model can be applied to new data, whether the user must modify the code manually, whether the code is automated to work by simply providing a data path, or whether there is a user interface that mediates the interaction between the user and the underlying code.

These aspects are particularly valuable for two distinct audiences. For non-specialists aiming to predict new interactions, our work functions as a practical guide, offering a clear comparison of available approaches, their requirements, and their trade-offs. This empowers researchers to select methods that best align with their objectives, data availability, and prior knowledge, thus reducing trial-and-error and accelerating experimental pipelines. For AI specialists, our analysis provides concrete insight into accessibility-oriented design choices, highlighting which practices have effectively broadened adoption beyond the expert community. Finally, our study not only maps the current state of the field but also acts as a catalyst for translating technical innovation into impactful real-world applications.

## Materials and methods

### Related works

This section discusses secondary studies directly related to this mapping. These studies can be divided into two groups: those on RPI prediction and those dealing with PPI. The first study is by Horlacher *et al*. [82], which presents RPIs from 2015 to 2022. For each study, the year, the dataset used for validation, the input to the model, the model used for prediction, and the type of prediction (classification or regression) are extracted. In addition, information about the datasets is reported, including the year, the first study to report it, and a description.

Still in the context of prediction of the RPI, Colantoni *et al*. [83] provides a comprehensive overview of the topic. This study focuses mainly on the various versions of catRAPID for *in silico* prediction, mentioning a few other similar tools. At the same time, *in vitro* and *in vivo* interaction discovery techniques, such as fluorescence anisotropy and X-ray crystallography, are reported.

Referring to tools related to PPI interactions, the first study is by Xiaotian *et al*. [78], which analyzes studies developed between 2010 and 2020, reporting the year, feature descriptor extraction, model, validation type, and URL of the application for each study. The description extraction techniques used in these tools, along with the prediction models employed and the reference datasets found, are briefly discussed.

Another secondary study related to PPI prediction is by Khatun *et al*. [79], which specifically focuses on techniques developed between 2001 and 2022 that use deep learning (DL) as a predictive model. For each tool, data such as year, input type, feature descriptor extraction, predictive model, training environment, validation datasets, and their associated performances are extracted.

Another important study was carried out by Mingda *et al*. [80], which reported research conducted between 2019 and 2023 that used some type of GNN for the prediction of PPI. In this study, data such as the year, the specific algorithm used, the validation dataset, and the availability of the code are reported for each tool. Finally, the last study by Xian *et al*. [81], which presents some tools and datasets for PPI modeling, with its main contribution being a thorough discussion of the most commonly used feature descriptor extraction techniques in this area of study.

In summary, these authors focus mainly on the technical aspects of the analyzed studies, such as the feature extraction, predictive models, and validation datasets. These aspects are essential to understand what has already been explored concerning technical aspects, in addition to exposing the current gaps in the approaches. In contrast, the proposed mapping seeks to understand aspects of the availability and accessibility of these tools. These aspects are relevant because, for a researcher in the biological field, there may be no practical difference between using a random forest (RF) or a multilayer perceptron, as long as both offer similar performance.

However, factors such as the availability of a user interface, which eliminates the need to directly manipulate code, or the ability of an approach to predict binding regions, can strongly influence the choice of technique to be employed. Thus, in addition to performance, complementarity reports and additional functionalities also become decisive in the decision-making process. In summary, this research seeks to complement existing secondary studies by providing a comprehensive overview of the state-of-the-art.

### Mapping protocol

Systematic reviews and mappings are mainly motivated as vehicles to rigorously gather evidence on a given topic systematically that ensures auditability and reproducibility [23]. In this section, the protocol used will be discussed, following the standard of Kitchenham [24]. This methodology proposes dividing the mapping into three main stages: planning, execution, and reporting, with several substeps [25].

#### Database and search string

For the proposed mapping, two databases were selected: Scopus and PubMed. Using titles, abstracts, and keywords, studies published in English in journals were searched in these databases with the following search string: (“PPI” OR “protein interaction” OR “RPI” OR “RNA interaction” OR “LPI” OR “lncRNA interaction”) AND (“machine learning” OR “deep learning” OR “link prediction”) AND (“tool” OR “framework” OR “toolkit” OR “package” OR “web server”). These search strings were applied in February 2026.

#### Inclusion and exclusion criteria

The following subsection outlines the criteria used for the inclusion and exclusion of studies in this work, as shown in Table 1. These criteria are necessary to ensure the reliability and relevance of the results obtained. Clearly defining inclusion and exclusion parameters enables a systematic and careful selection of studies, ensuring that only relevant and high-quality data are considered in the analysis.

**Table 1:** The inclusion and exclusion criteria outlined below were applied to all studies retrieved through the search string

No.	Inclusion	Exclusion
1	Describes a tool for predicting interactions between proteins, RNAs, or RNA–protein.	Does not describe a tool for predicting interactions between proteins, RNAs, or RNA-protein.
2	Describes a tool for predicting interactions between biological sequences, compared with at least one other tool on more than one dataset.	Does not describe a tool for predicting interactions between biological sequences, compared with at least one other tool on more than one dataset.
3	Provides access to the described tool (source code or web server) for readers to reuse the tool.	Does not provide access to the described tool (source code or web server) for readers to reuse the tool.
4	Published in a journal.	Not published in a journal.
5	Not published as an abstract only.	Published as an abstract only.
6	Written in english.	Not written in english.
7	It is not an older version of another study already considered.	It is an older version of another study already considered.
8	Not an older version of another study already considered.	Is an older version of another study already considered.
8	A primary study.	Not a primary study.
9	The study was accessible.	The study was not accessible.

#### Implementation of the review protocol

Initially, after searching the interaction databases, 2506 unique studies were identified. After screening the abstracts, only 275 studies were considered eligible. A subsequent full-text review revealed that 172 of these studies focused on predicting interactions between biological sequences, specifically those meeting the first and fourth to ninth inclusion criteria.

Of this group, in a subsequent review, 86 studies were discarded based on exclusion criteria two and three. Specifically, 25 studies were eliminated by the second criterion: 17 for not performing comparative validation with other studies, which means that there was no performance comparison with at least one independent study, and 8 for using only one dataset during the validation process, suggesting that the tool was developed specifically for that dataset. Furthermore, 61 studies were excluded by the third criterion for not providing access to their code or means of access.

This highlights that half of the interaction prediction studies found were discarded, with most being methodological descriptions, i.e. they did not provide access to the algorithm that generated the results. This excludes non-programmers and ML specialists from using these approaches. The 86 studies that passed were evaluated for the purpose of writing this mapping (See summary and trend analyses in the Supplementary Material).

## Results: inputs, interpretability, and decision-making

In this section, we focus on studies that employ input strategies beyond the primary structure and provide outputs that extend beyond simple interaction predictions. Specifically, we discuss tools that utilize alternative sources of information for molecular characterization and those that generate additional reports to complement the prediction results. The characteristics and functionalities of these tools are examined to highlight how they enhance the performance of the model and the decision-making process.

### Results

#### Inputs

Several tools were identified to predict interactions between biological sequences; however, they employ different strategies, and in this section, we discuss the diversity of approaches regarding the input used by these models. In Supplementary Fig. 5a, it is evident that the vast majority of tools rely on the primary structure in the FASTA format to characterize the sequences. In other words, the descriptors for characterizing proteins and RNAs are extracted from the amino acid or nucleotide sequences that compose these molecules, making this the most common strategy adopted in the state-of-the-art.

However, not all studies rely solely on this information for descriptor extraction; other relevant sources of information are also used. Understanding which information is used to make predictions involves recognizing the data considered by the models and what is required in terms of input to apply them in new scenarios. In the following, we discuss individually the input strategy adopted by each of the models listed in Table 2.

**Table 2:** Compilation of tools with inputs different from the primary structure

Tools	Year	Alternative inputs
Network-based PPI [26]	2019	Only known interactions
GOSeqPPI [27]	2020	Gene Ontology
PPI-RCC [28]	2020	3$\underline{\circ }$ structure
PrismNet [29]	2021	icShape
TransformerGO [30]	2022	Gene Ontology
Struct2Graph [31]	2022	3$\underline{\circ }$ structure
GKLOMLI [32]	2023	Expression profile
HDRnet [33]	2023	icShape
HIGH-PPI [34]	2023	3$\underline{\circ }$ structure
DeepAraPPI [12]	2023	Gene Ontology
FMSRT-LPI [35]	2024	Gene Ontology
HiGPPIM [36]	2024	SMILES notation
INTREPPPID [13]	2024	Gene Ontology
LPI-KCGCN [37]	2024	Gene Ontology
PPI-BAN [38]	2025	3$\underline{\circ }$ structure
HI-PPI [39]	2025	3$\underline{\circ }$ structure

First, we have the network-based PPI [26], which uses only known interactions to predict missing interactions in the network. It extracts feature descriptors from the input network and predicts new links between proteins in that network. Next, HiGPPIM [36], which does not use the primary structure in FASTA format with nucleotide or amino acid sequences, but rather uses these sequences in the notation SMILES (Simplified Molecular Input Line Entry System). This notation is a textual representation of chemical structures processed by a GAT network that extracts feature descriptors and characterizes each biological sequence.

Another approach used in DeepAraPPI [12], GOSeqPPI [27], TransformerGO [30], INTREPPPID [13], LPI-KCGCN [37], and FMSRT-LPI [35] is the GO notation. This notation consists of a standardized system to describe the attributes of genes and gene products, such as molecular function, cellular component, and biological process. GO input information is processed by various approaches, in DeepAraPPI [12], TransformerGO [30], and INTREPPPID [13], such as node2vec. In FMSRT-LPI [35] and LPI-KCGCN [37], a correlation matrix is used. Finally, in GOSeqPPI [27], natural language models such as BERT.

Furthermore, the tools PPI-RCC [28], Struct2graph [31], HIGH-PPI [34], PPI-BAN [38], and HI-PPI [39] require the tertiary structure of proteins. These tools utilize the arrangement and connection between different amino acids to extract information using techniques such as GCNs or residue clustering classes (RCCs). In these approaches, the spatial arrangement of the submolecules that compose the protein is considered, allowing for a more detailed characterization on the basis of the 3D structure of the proteins.

Finally, other tools use input related to *in vivo* sequence information. The first tool in this sense is GKLOMLI [32], which uses the expression profile of RNA sequences in living cells along with their biological function, processing these inputs through a similarity matrix to characterize RNAs. We also have PrismNet [29] and HDRnet [33], which use experimental RNA information from icSHAPE-type experiments as input for predicting new RPIs, where icSHAPE is an *in vivo* technology for determining information about the secondary structure of RNAs by measuring the flexibility of the bases that compose it [29, 40], improving the structural representation of these molecules.

#### Interpretability and decision-making reports

Some identified studies, in addition to predicting new interactions, also provide complementary reports that add relevant information to the investigated problem. These reports were initially classified into two categories: interpretability, which includes graphs and tables aimed at explaining the models’ decisions (that is, justifying the results obtained), and decision-making, which gathers additional information related to predicted interactions, helping to select pairs for further investigation. Such approaches contribute to increasing the transparency of the decision-making process and provide a greater context for the identified interactions, thereby expanding the set of information available to support subsequent decision-making.

Table 3 lists the studies that include interpretability and decision-making reports with these functionalities implemented in the reported code, making them available to users for new datasets. It is important to note that not all studies that present interpretability graphs provide these functionalities in their code, and therefore, they are not included in this section.

**Table 3:** Summary of interpretability and decision-making found in the state-of-the-art

Tool	Year	Task	Interpretability	Decision making
PrismNet [29]	2021	RPI	–	binding site
TransformerGO [30]	2022	PPI	feature importance	–
ProteinPrompt [41]	2022	PPI	–	performance graph
deepHPI [42]	2022	PPI	–	interaction network
Struct2Graph [31]	2022	PPI	–	key residues
HIGH-PPI [34]	2023	PPI	feature importance	binding site
HDRNet [33]	2023	RPI	–	binding site and interaction network with GO
Deep-HPI-pred [43]	2023	PPI	–	interaction network with GO
SGPPI [44]	2023	PPI	–	binding site
LPI-MFF [45]	2024	RPI	LIME and SHAP	–
INTREPPPID [13]	2024	PPI	GO enrichment	–
ProRNA3D-single [46]	2024	RPI	–	3D interaction
DNE [47]	2024	PPI	–	function prediction and interaction network
BioPrediction-RPI [7]	2024	RPI	SHAP	–
ESM2_AMP [48]	2025	PPI	–	key residues
PPI_BAN [38]	2025	PPI	–	binding site
LncPTPred [49]	2025	RPI	–	binding site
MsipNet [50]	2026	RPI	–	key residues
AttnSeq-PPI [51]	2026	PPI	–	interaction network

An initial, simple, and common technique for interpretability is feature importance, in which metrics are assigned to each feature, allowing them to be classified and identifying the most influential in the final decision process [52]. In this regard, feature importance techniques that highlight the physicochemical properties of amino acids that are most relevant for discriminating classes in HIGH-PPI were found [34]. Furthermore, TransformerGO [30] also utilizes feature importance to highlight the 30 most frequent GO terms used during the classification task. Similarly, INTREPPPID [13] returns importance measures to classify each GO term. Finally, tools such as DeepPPAPred [53], MaTPPI [54], and SELPPI [55] discuss the importance of their feature descriptors, although this functionality is not included in the code. This can be leveraged if the user uses the same dataset as the study, but it is not available for new experiments.

Model interpretability techniques, such as LIME [56] and SHAP [57], are used to explore the decision-making process, allowing detailed visualization of the contribution of each feature descriptor. These methods are integrated into the code in tools such as LPI-MFF [45], SELPPI [55], and BioPrediction-RPI [7]. In addition to feature importance plots, these methodologies offer other types of visualizations, such as graphs that relate the value of a feature descriptor in a specific sample to its relative contribution to classification into one of the classes, showing, e.g. how high values of a feature descriptor are associated with a positive class.

To enhance user decision-making, some tools provide complementary outputs. For instance, the Web server of ProteinPrompt [41] offers graphs with metrics related to the threshold of pretrained models. When predicting a pair, it provides only the score from 0 to 1, allowing the user to select the most appropriate threshold. Thus, a threshold above 0.50 would result in a more precise and less sensitive model, while a lower threshold would increase sensitivity, even at the cost of precision.

This is useful if the user needs to predict only a few positive interactions and wants to increase the decision threshold for a more precise model. Alternatively, if the user aims to capture a minimum percentage of all positive interactions, they can choose the corresponding threshold to achieve the desired sensitivity, even if it leads to a reduction in model precision.

Regarding decision-making reports, some tools, such as deepHPI [42], DNE [47], and AttnSeq-PPI [51], display newly predicted pairs within an interaction network. Certain tools, such as Deep-HPI-pred [43] and HDRNet [33], further enrich this network by including the GO annotation of each node. This type of visualization enables users to observe connection patterns in the network, providing valuable guidance. Similarly, DNE [47] also predicts the function of each protein, providing deeper insights into the nature of the predicted interactions.

Finally, some tools also offer predictions of key residues, such as Struct2graph [31], ESM2_AMP [48], and MsipNet [50], and binding regions, as implemented in PrismNet [29], SGPPI [44], HDRNet [33], LncPTPred [49], and PPI_BAN [38], all of which are based solely on the primary structure as input. These predictions are essential for guiding users to critical regions of the molecules that warrant special attention. For instance, by identifying important residues or binding sites, users can focus their efforts on investigating cavities where a drug may bind more effectively. Similarly, ProRNA3D-single [46] provides 3D visualization of protein–RNA interactions, allowing users to understand how the molecules interact in space.

### Web servers

**Figure 1:**
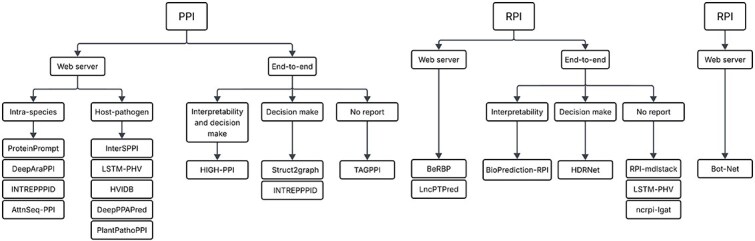
Tree diagram to summarize the main tools found, organized in relation to whether they are web servers or end-to-end frameworks and in relation to the types of reports available by model.

In Figure 1, we present a representation of the identified tools that are end-to-end or available as web servers. In addition, we show the subdivisions by task (PPI, RPI, and RRI), together with key points of each approach, enabling a quick filtering of the literature to identify the most suitable tools for each application.

One common approach to make these tools available is through web servers that host algorithms capable of performing new predictions, providing users with an accessible online interface (see Fig. 1). Predominantly, these servers employ pretrained models to predict new interactions without offering the option to fine-tune the model using user-provided data. To use these servers, users need to input the interactions they wish to predict along with the primary structure of all the biological molecules involved. This prior training establishes the specific context in which the models were developed and validated, which means that they are tailored to a particular organism or group of organisms.

**BerRBP** [58] is the oldest web server identified in this mapping. It offers a platform for the prediction of RPI, specifically using human biological sequences and employing an RF classifier. This server provides numerous position weight matrices (PWMs) for the user applications, along with a customization option. PWMs serve as representations of RNA motifs and are essential to identify and quantify the potential binding affinity of sites within the candidate RNA sequence.

**InterSPPI** [59] is the only web server released in 2020 that focuses on PPI, with a specific emphasis on host–pathogen interactions. It offers three pretrained models: one for human–virus interactions, another for human–bacteria interactions, and a third for Arabidopsis thaliana–pathogen interactions. This setup allows users to select the model that best suits their research needs. One limitation reported by the authors is that the server only accepts protein sequences with a minimum length of 35 amino acids.

**Bot-Net** [60] is a web server focused on RRIs, and it is the only one in the mapping that supports both training a model based on user-provided known interactions and using pretrained models for human lncRNA–miRNA interactions. In training mode, the server requires users to input not only the known interactions but also parameters such as the k-value for k-mers, the number of folds, the number of epochs, and the learning rate for the CNN-based classification model. This flexibility allows users to customize and fine-tune the training process, but also demands manual configuration to select the most suitable hyperparameters.

**LSTM-PHV** [61] is a web server dedicated to the prediction of novel PPIs, focusing specifically on human–virus interactions. It uses an LSTM model combined with Word2Vec embeddings for prediction. In this tool, the user inputs the protein pairs to be evaluated, and the model outputs the interaction probability for each pair.

**HVIDB** [62] is another web server dedicated to the prediction of human–virus PPIs. In addition to predicting novel interacting pairs, this server is integrated with a comprehensive database containing 48 643 interactions, involving 7796 human proteins and 2104 viral proteins. If the queried protein is present in the database, the server provides information about it within a complex interaction network, along with structural and GO annotations of the proteins involved in that network.

**ProteinPrompt** [41] is a web server for PPI prediction, designed to identify potential ligands for a user-specified protein, using proteins from humans, mammals, vertebrates, or metazoans. The server outputs a score for each tested pair, which can be predicted using either an RF model, a convolutional neural network (CNN) model, or a consensus between both methods. Additionally, the server provides performance plots, including balanced accuracy, F1 score, precision, sensitivity, and specificity across various thresholds for both models. This allows users to choose their threshold, enabling them to opt for, for instance, a more precise but less sensitive model, or a more sensitive but less precise one, thus supporting more informed decision-making.

**DeepHPI** [42] presents a set of pretrained models on PPIs, including: human–bacteria, human–virus, plant–pathogen, and animal–pathogen. The tool predicts all interactions among the proteins provided by the user and reports the results in a table listing the detected interactions. In addition, it offers interactive network visualization, allowing users to explore the predicted interactions and export a figure of the network.

**DeepPPAPred** [53] works with prediction between human proteins and pathogens, offering several pretrained models classified by protein function. The available models include antigen-antibody, enzyme-containing, receptor-containing, G-protein-containing, other enzymes, and miscellaneous. Unlike other tools, it uses regression models to predict the affinity between protein pairs, returning the free binding energy, dissociation constant, and indicating whether the binding affinity is low or high.

**DeepAraPPI** [12] is a PPI prediction server that supports two model organisms, each with four predictive submodels. Among these submodels, the convolutional recurrent neural network (RCNN) and logistic regression (LR) stand out, both using the primary structure of proteins as input. Additionally, the server includes the GO2Vec model, which can predict PPIs based on GO. However, to use this model, it is necessary for the proteins involved to have GO information available.

**INTREPPPID** [13] is a PPI prediction server with a pretrained model on human interactions, allowing predictions against proteomes of organisms such as human, mouse, zebrafish, fruit fly, nematode, and thale cress. After predicting the target protein, the server provides the probability of interaction with each protein from the chosen proteome, along with a histogram of the probabilities. In addition, it offers enrichment in terms of GO and their associated importance.

**LncPTPred** [49] is a server designed for RPI prediction. Specifically, the model was trained using 88 characterized proteins, enabling the prediction of interactions between any novel lncRNA and one of these proteins, and returning the putative binding regions between the molecules, when such interactions exist.

**PlantPathoPPI** [63] is specifically focused on PPIs from plant–pathogen interactions. It is trained using a combination of several host–pathogen interaction databases, filtering for interactions involving only plant proteins and integrating them into a single unified dataset.

**AttnSeq-PPI** [51] is a web server that employs a two-channel hybrid attention mechanism that integrates global sequence features with contextual residue-level information extracted from protein sequences. The model leverages Prot-T5-XL embeddings to capture rich per-residue representations and utilizes self- and cross-attention to model interacting residues between protein pairs. Users provide protein sequence pairs as input, and the server returns interaction probabilities along with an interactive visualization of the predicted PPI network.

### End-to-end studies

This section analyzes studies that position themselves as end-to-end tools for predicting interactions between biological sequences (see Figure 1, also detailed in Supplementary Table 1). Among the $\sim$86 tools identified in the systematic mapping, only ten can be considered end-to-end solutions. These tools provide a continuous workflow, covering the entire process from descriptor extraction to final prediction, without requiring additional interventions or integration with external tools.

In total, five end-to-end tools focused on PPI tasks and three on RPI tasks were identified. Most of these tools, except for two, offer comprehensive documentation to help users apply them to new datasets. Although all of them require the primary structure of molecules as input, three models also provide alternative options, such as tertiary structure and experimental data, which enrich molecular characterization. During the prediction phase, most are relying on black-box models, and about half of the tools include additional reports to improve the interpretation of the results.

**Rpi-mdlstack** [64] is a tool for RPI prediction that employs a bioinspired feature descriptor extraction approach, such as k-mer, based on the nucleotide frequency to characterize the biological molecule. The classification process is carried out in two stages: initially, the tool performs a preliminary classification using multiple models, such as a multilayer perceptron, RF, and a deep neural network. Subsequently, the decisions of these classifiers are combined using a support vector machine for the final decision, resulting in an approach that integrates both white-box and black-box models. However, the GitHub repository for the tool does not provide clear guidance on how to reproduce the experiments from the original study or apply Rpi-mdlstack to new datasets.

**HDRNet** [33] is a tool for RPI that leverages both the primary structure and the icSHAPE characterization of RNAs. The model extracts feature descriptors from both sources for the final prediction through a CNN, using BERT for the primary structure and one-hot encoding for the icSHAPE data. In addition, HDRNet performs various analyses, such as feature importance assessment and the identification of important regions in each molecule.

**ncrpi-lgat** [65] is a tool for RPI that uses node2vec for the extraction of feature descriptors from primary sequences and also applies a Subgraph Extraction and Aggregation Layer (SEAL) to generate subgraphs, leveraging graph attention networks (GATs) to extract topological information. **TAGPPI** [66] is a tool capable of predicting PPIs using SeqVec embeddings (Sequence-to-Vector), derived from the ELMo model trained on UniRef50, as its main feature descriptor. After this, a multilayer perceptron is employed for the final prediction. However, similar to the previously mentioned tool, TAGPPI lacks clear documentation on how to preprocess each dataset used in the validation or how to apply the tool to new data.

**Struct2graph** [31] is a predictive tool for PPIs that leverages both the primary and tertiary structures of proteins. However, the tertiary structure does not need to be provided directly, as it is retrieved from the Protein Data Bank (PDB), which may be a limitation if the proteins are not available in that database. The tool uses an architecture based on GATs and graph convolutional networks (GCNs) to characterize proteins, followed by a feedforward neural network (FNN) for interaction prediction. Additionally, Struct2graph can identify and predict the amino acid residues it considers key to the interaction for each protein pair.

**HIGH-PPI** [34] is a tool for PPI that uses a GCN to extract information from the topology of the tertiary structure of proteins. In addition, it employs a graph isomorphism network (GIN) to capture additional feature descriptors from the known interaction network, performing the final classification based on this combined data. The user is required to provide the tertiary structure of all proteins, which can be retrieved from online databases or predicted using AlphaFold.

**INTREPPPID** [13] is a PPI method that incorporates orthology data through a quintuplet neural network, consisting of five parallel encoders with shared parameters. It combines a PPI classification task with an ortholog locality learning task, ensuring that orthologous proteins have closely located embeddings in Euclidean space.

**BioPrediction-RPI** [7] is an end-to-end ML framework for predicting interactions between biological sequences, such as non-coding RNA and protein pairs, without requiring specialized expertise. It performs feature engineering to represent the sequences using structural and topological attributes, which are organized into feature groups to train partial models. The decisions of these models are then combined into a final prediction, accompanied by an interpretability report, based on SHAP values.

## Discussion

This mapping considered several aspects of studies that focused on the prediction of interactions between biological molecules, with particular emphasis on the accessibility of the proposed tools. Such tools are essential for the discovery of novel interacting pairs as they enable the computational testing of millions of combinations, significantly accelerating the process of identifying relevant biological interactions.

### General trends in the area

According to our trend analysis, in the Supplementary Material, the number of tools that focus on predicting interactions between biological molecules has been increasing since 2020. When analyzing their quantity and categories, we observe that most studies focus on PPI tools, followed by RPI and, lastly, RRI. Furthermore, the findings show that the available tools are generally provided either as web servers or with accessible source code, whether they are end-to-end or not.

When examining documentation, this study classified the results into three categories: documentation to apply the tool to new data, documentation to reproduce experiments from the original article, and no documentation. When this information from Supplementary Fig. 2 is combined with Supplementary Fig. 3b, it becomes evident that, despite the growth in the number of tools targeting these challenges and the emergence of some automated approaches, there has been a decline in the production of adequate documentation for their use. This scenario suggests a potential gap in user support that may limit both the accessibility and reproducibility of these tools, particularly in contexts that require adaptation to new data, such as studying interactions in previously unexplored species.

Finally, it is observed that most of the studies included in this mapping correspond to tools accessible only through source code, and within this category, the majority lack any form of documentation. Furthermore, approximately half of the studies focused on interaction prediction were excluded because they did not provide any form of access, not even undocumented source code. This scenario is highly unfavorable for the practical application of these tools, as most require prior knowledge in AI or programming, whether to implement the code when it does not exist, understand it when poorly documented, or adapt it when it is not fully automated.

### Inputs and outputs

Regarding the required inputs, the proposed tools rely heavily on the primary structure of sequences as the main source of molecular characterization. To extract relevant information, these approaches often combine mathematical descriptors with black-box representations such as embeddings. Predominantly, they employ DL-based predictive models, aiming to achieve higher accuracy.

Although there are various input types beyond the primary structure in the FASTA format, many of which can predict new interactions with robust performance, these approaches remain underexplored. This may be because some input types are highly specific, resulting in tools that are only applicable to limited contexts, such as those that use molecular expression profiles. Another relevant factor is the computational cost, as seen in the processing of 3D structures for thousands of proteins, which can make large-scale usage impractical.

Despite these limitations, additional data sources are complementary to studies of biological sequence interactions and are useful across different stages of multiple research pipelines. For example, the analysis of protein tertiary structure enables the extraction of structurally relevant information that facilitates the understanding of protein mechanisms of action [67]. In this context, tools such as HIGH-PPI [36] leverage structural information both to predict interaction labels and to approximately identify binding regions. This strategy provides more detailed insights into the domains involved in molecular interactions, contributing to a deeper understanding of the functional behavior of target proteins.

Complementarily, when the objective is to assess the impact of proteins on pathways and phenotypes, the use of GO data allows a better understanding of how proteins are regulated and participate in biological processes [68]. Accordingly, studies that integrate GO information, such as INTREPPID [13], offer a distinct advantage by explicitly modeling the influence of functional annotations on prediction decisions. Although the collection and integration of such complementary information can be labor-intensive or restricted to specific experimental contexts, these approaches enable analyses that go beyond simple binary interaction classification, substantially enriching the functional interpretation of protein interaction networks, which represents a key strength of these models.

As a result, the primary structure remains the predominant input format, as it is widely available and computationally more accessible. This scenario reveals a gap in the state-of-the-art concerning the use and integration of diverse input types, indicating the need for strategies that incorporate new data efficiently, without compromising applicability or requiring excessive computational power. Overall, a key recommendation to improve versatility is to provide multiple versions of trained models, including one that relies solely on primary sequence information and another that incorporates complementary inputs. This design would allow for a systematic evaluation of the performance gains achieved by integrating additional data sources, thereby incentivizing the acquisition and use of such complementary information.

Regarding the outputs of these tools, some studies include interpretability reports, while others focus on decision-making. The first type relies mainly on strategies derived from feature–importance analyses. In contrast, decision-making-oriented studies vary between predicting aspects related to the interaction interface and incorporating functional information about the proteins involved. This contrasts with traditional approaches, which are grounded in docking techniques and thermodynamic principles derived from physical and biological concepts, where the underlying mathematical models can be explicitly analyzed, as opposed to learning from examples. In such approaches, tools frequently produce outputs in the form of interaction networks accompanied by Gene Ontology annotations, as in STRING [69], perform the removal of intrinsically disordered regions to improve structural modeling, as in ClusPro [70], or enable 3D visualization of predicted molecular complexes, as in NPDock [71].

In general, the lack of auxiliary reports remains underexplored, despite their potential to provide valuable information to users. This gap highlights that many tools primarily prioritize model performance, often neglecting the provision of insights that could enrich decision-making. The inclusion of interpretable reports would significantly contribute to understanding the model’s decision-making process, identifying its limitations, and planning experiments, thus assisting users in subsequent stages of their research. In this systematic mapping, we identified studies that employ alternative inputs beyond the primary structure, as well as complementary outputs capable of addressing specific demands during application. This compilation of non-conventional approaches provides quick access to such information, supporting the selection of the most suitable methodology.

### Web servers scenario

First, web servers represent the most accessible class of tools identified in this study, as they offer an intuitive interface and typically operate with pretrained models. In this way, users only need to upload the interactions of interest with the primary structure of all biological molecules and wait for the predictions to be generated, without requiring manual adjustments, advanced technical knowledge, or programming skills.

It is interesting to note that among the set of pretrained models for PPI prediction, there are those capable of predicting interactions between proteins of the same organism, such as human proteins [41], mammalian [41], vertebrate [41], and metazoan proteins [41].

Taking into account this, some models have been tailored to PPIs between proteins of different species, covering important host–pathogen groups, such as human virus [42, 53, 59, 61, 62], human bacteria [42, 53, 59], animal pathogen [41, 42], and plant pathogen [42, 59]. These models provide a solid foundation for the analysis of interspecies interactions, which is essential to understanding the pathogenic mechanisms and host–pathogen dynamics.

However, considering the variability of species across the three major domains, Bacteria, Archaea, and Eukaryotes, it is evident that the studies identified do not fully cover this biodiversity. An example of this limitation is the absence of models specifically trained to address interactions between plant proteins. Currently, there is only one model designed for plant–pathogen interactions, which means that there are no tools dedicated to understanding interactions exclusively between plant proteins. This gap hinders advances that could enhance our knowledge of plant biological mechanisms and contribute to genetic improvement, which is of great interest and relevance for agricultural production.

A similar situation is observed for predicting the interactomes of viruses and bacteria, which also represents a significant gap. Tools in this area could offer valuable insights into the metabolism of infectious organisms and support the development of therapeutic or preventive strategies. These gaps underscore clear opportunities to create more targeted models in these research domains. In this context, there is also no pretrained model available to handle any type of interaction involving organisms from the taxonomic domain of Archaea, as well as important groups within Eukaryotes, such as fungi.

In contrast, the STRING interaction database [68] provides interaction networks across a broad taxonomic spectrum, including 128 species within the *Plantae* group, 663 species from *Fungi* group, and 457 species from the domain *Archaea*. These data could be leveraged to validate model performance across a wider diversity of species and taxonomic groups, thereby providing evidence that proposed models can be applied beyond a narrow set of well-characterized organisms.

Nevertheless, even with the variety of pretrained models available, there is a clear lack of web servers that allow users to train a new model based on their known interactions. This functionality would be extremely valuable for predicting new interactions using a model tailored to a specific problem, such as a particular virus species. It would enable researchers to adapt the model to their specific needs and explore previously uninvestigated interactions with high performance.

Moreover, it is evident that in the field of RPI prediction, pretrained models are predominant, and the lack of options for training context-specific models represents a significant limitation. A particularly concerning aspect is that the available pretrained models are restricted to interactions between human RNAs and proteins [58], severely restricting their applicability in various biological scenarios. This lack of diversity compromises the use of these tools in applications such as RNA-based vaccine development, where the RNA of interest may be synthetic or non-human. Since these models have been trained exclusively on human RNAs, there is no guarantee that their predictions will be accurate for other types of RNA, thus limiting their impact.

The RRI prediction server [60] offers a pretrained model for human RNAs and supports training new models with data from other organisms. This is feasible given the smaller number of studies in the field, suggesting a reduced user base, and because RNAs are generally smaller and less complex, lowering computational costs and making server maintenance sustainable. Bot-Net is not fully automated but allows adjustments to hyperparameters such as k-mer size and training epochs. While this adds versatility, it can challenge users without ML experience, as misinterpreting performance metrics may cause overfitting. The tool also provides no guidelines on dataset quality, potentially limiting model effectiveness.

This is the only scenario where training new models is possible. For other interaction types involving more complex molecules, the high computational cost may be a limiting factor, and very large datasets could overload the server, making this option infeasible. This restriction is also linked to method validation: although the approach performed well with the original authors’ data, its robustness with different training datasets is unproven.

If users provide imbalanced or poorly representative data, such as interactions between only very similar molecules or molecules very different from the target, the model may not generalize well, compromising prediction quality. In such cases, only the authors’ pretrained models are available, ensuring reliable predictions.

Web servers are highly accessible for predicting new interactions but are limited by models tailored to specific contexts. Developing efficient features and lightweight models with strong performance could enable online training, combining remote accessibility with greater customization. In general, Fig. 1 can be used as a guide for model selection. The process begins by choosing the task of interest (PPI, RPI, or RRI), followed by selecting between a web server or an end-to-end model. If an end-to-end model is chosen and a specific type of reporting is required, the corresponding branch of the tree can be selected accordingly; otherwise, all available options are considered valid.

### End-to-end and non end-to-end scenario

On the other hand, there are end-to-end tools, developed to allow the adjustment of new predictive models based on the data provided by the users themselves. In this case, users need to input known interactions for model training, followed by the target interactions they want to predict. However, these approaches generally do not offer a graphical interface, requiring input through code, even if minimal. In this case, the user defines the context of the predictive model. This contrasts with web servers, which, despite using fixed models, provide a user-friendly interface that makes it easier for non-technical users to operate.

First, in the end-to-end approach, we observe that most studies use only the primary structure to characterize molecules. However, within this group, a considerable fraction incorporates other sources of information, enhancing the richness of the molecular representation. As an example, HDRNet [33] uses experimental icSHAPE data, while Struct2Graph [31] and HIGH-PPI [34] consider the tertiary structure. This approach can be especially useful for users who have access to such additional data, as it allows for a more detailed characterization of the molecules. Furthermore, these tools provide auxiliary outputs that offer more information about the problem, such as the identification of binding regions. Thus, although these methods require more specific inputs, they also enrich the interpretation of the results, making them valuable options for scenarios where additional structural information is available.

Furthermore, we find that end-to-end approaches typically use black-box predictive models to make predictions. An exception is Rpi-mdlstack [64], which combines black-box prediction with a white-box model, although the final decision remains black-box. Another exception is BioPrediction-RPI [7], which stands out by using decision tree-based models exclusively for classification, ensuring greater transparency in the process. Additionally, among all the tools analyzed, only two have integrated interpretability techniques: HIGH-PPI [34], which provides analysis of physicochemical properties characteristics, and BioPrediction-RPI [7], which offers SHAP-based graphs, including the importance of each physicochemical characteristic descriptor. This highlights that to date, the concern with interpretability remains limited, present only in two models.

On the other hand, end-to-end tools face the main challenge of lacking user-friendly interfaces, as they are accessed through the source code, usually with documentation to assist. Although all modeling is automated, this limitation can hinder the use of researchers without programming expertise, restricting their potential impact. In end-to-end scenarios, no tool was found that automates the process of developing the dataset, requiring the user to provide known interactions for model adjustment. This represents a significant limitation. Furthermore, no studies were identified that provide clear documentation on the expected characteristics of the input data or best practices for constructing the training set.

This absence is concerning, as it allows the user to input unbalanced data, misaligned with the final prediction context, or of different sizes compared with those used during model validation. In other words, the model may end up being trained with examples that do not reflect the patterns of interactions the user truly wants to predict. Therefore, automating the development of the dataset, tailored to the interactions the user wishes to predict, could represent a promising advancement. This would ensure not only more robustness and consistency in the generated models, but also greater autonomy and performance of the tools in real-world application contexts.

In contrast, studies that propose non-end-to-end methods, although representing the less accessible class for users, play a fundamental role in advancing the state-of-the-art. These works are generally focused on investigating new methodologies and approaches for interaction prediction, with an emphasis on deepening the understanding of the problem, rather than necessarily providing tools for end users. This constitutes the majority of studies, unfortunately showing that most articles found do not prioritize accessibility and reproducibility but focus solely on developing new approaches instead of addressing both.

### Classical approaches vs. machine learning

In parallel, the prediction of biomolecular interactions represents a well-established research field, with numerous studies developed long before the emergence of ML models. To contextualize these traditional approaches, we present below a representative selection, highlighting their underlying mechanisms (see a summary of the methodology of these approaches in the Supplementary Section “Classical Approaches description”). All of these solutions are available as web servers that remain online and require only the structural information of the molecules under study as input.

First, we consider the STRING database [69, 72], which integrates PPIs data inferred from multiple sources of evidence, including gene neighborhood, gene fusion, phylogenetic co-occurrence, experimentally derived interactions, and text mining of the scientific literature. Beyond integrated evidence databases, there are computational techniques based on physical docking, such as ClusPro [70] for predicting PPIs, HDOCK [73] for both PPIs and RPIs, and NPDock [71] for RPIs. In general, tools such as ClusPro [70] and HDOCK [73] rely directly on 3D structures obtained from the Protein Data Bank (PDB), whereas HDOCK [73] and NPDock [71] also allow the inclusion of additional information, such as specific regions where binding is expected to occur. Still within the context of RPIs, RBPmap employs a sequence- and motif-based approach to predict RPIs binding sites, enabling the identification of potential interactions between RNAs and proteins in genomes such as those of humans, mice, and *Drosophila melanogaster*.

For RRIs, there are also methods based on thermodynamic models, such as RNAplex [74], IntaRNA [75], and RNAup [76]. These tools provide different levels of experimental and computational control. For instance, RNAplex [74] allows the adjustment of system temperature, whereas RNAup [76] enables the definition of the minimum expected alignment length between RNA molecules. Additionally, many of these approaches expose several computational hyperparameters, including the number of poses generated in clustering-based docking procedures, the use of GPU or CPU resources, and the specification of initialization seeds to ensure reproducibility.

Overall, these traditional approaches are generally more accessible than modern ML-based methods, as many of them have been available online for decades through stable web servers. Despite being provided through graphical interfaces, these platforms typically include detailed documentation describing their usage, input requirements, and parameter configurations. Moreover, they are widely cited and well referenced in the scientific literature, reflecting their long-standing adoption and reliability within the research community. In this context, modern ML techniques can benefit from these traditional methods, both by adopting similar standards of documentation and accessibility, and by enabling the development of hybrid models that integrate established biological knowledge with data-driven learning approaches.

## Conclusion

Advances in molecular biology and the advent of high-throughput sequencing have exponentially increased the volume and complexity of biological data, intensifying the need for effective computational tools to predict interactions between biological molecules. Our systematic mapping highlights a significant growth in research focused on interaction prediction since 2020, reflecting intensified efforts driven by recent global health challenges.

However, despite this surge, accessibility remains a critical bottleneck: the majority of available tools require substantial expertise in ML or programming, with only a minority providing user-friendly Web servers or fully automated end-to-end tools. In addition, the prevalent focus on predictive performance often comes at the expense of interpretability and decision-making, as few studies provide auxiliary interpretability reports or comprehensive documentation to support broader adoption and reproducibility. This gap limits the practical utility of many promising approaches, especially for biologists and non-expert users who could greatly benefit from these technologies.

Our analysis further reveals a strong reliance on primary sequence information for modeling, with alternative input types and integrative approaches still largely underutilized, likely due to computational costs and data-specificity challenges. In particular, the coverage of diverse biological domains remains uneven, with limited models addressing interactions beyond human proteins, such as those in plants, fungi, archaea, or diverse pathogens, representing key opportunities for future research. It is widely observed that most pretrained models evaluate their performance primarily on human interactions or exclusively on well-studied model organisms, such as *Mus musculus, Arabidopsis thaliana*, and *Drosophila melanogaster* (the fruit fly) [77]. This practice restricts the evidence of model applicability to a limited set of species.

In summary, this work underscores the urgent need for the development of accessible, interpretable, and well-documented tools that combine robust predictive power with user-centric design. Facilitating the integration of such tools into biological research workflows will accelerate discoveries, promote reproducibility, and democratize the use of ML in the life sciences, ultimately driving transformative progress in understanding biological interactions and their implications for health and disease.

**Key Points**

- A complementary analysis of protein–protein interaction (PPI), RNA–protein interaction (RPI), and RNA–RNA interaction (RRI) prediction studies, extending previous secondary analyses that primarily focus on model design choices.
- A comprehensive mapping of key usability aspects across PPI, RPI, and RRI tools, including required inputs, output formats, and tool availability, providing practical guidance for biologists in selecting suitable methods.
- An overview of emerging trends in accessibility and usability within biomolecular interaction prediction tools.
- A critical reflection on current design practices, highlighting the persistent gap between methodological innovation and real-world applicability.

## Supplementary Material

bbag226_Supplemental_File

## Data Availability

No data were used in this study.
