# Supplementary material for: Accessibility in proteins and RNAs interactions prediction with machine learning: are we overlooking non-experts?
Source: Brief Bioinform. 2026 May 11;27(3):bbag226. doi: 10.1093/bib/bbag226 (PMC13158124; doi:10.1093/bib/bbag226)
Supplement: bbag226_Supplemental_File [file bbag226_supplemental_file.pdf]

Journal Title Here, 2022, pp. 1–7

doi: DOI HERE
Advance Access Publication Date: Day Month Year
Paper

PAPER

(Supplementary Files) Accessibility in Proteins and RNAs Interactions Prediction with Machine Learning: Are We Overlooking Non-Experts?

Bruno R. Florentino,[1,3] Robson P. Bonidia[1,2,3]* and André C. P. L. F. de Carvalho[1,3]*

[1]Institute of Mathematical and Computer Sciences, University of São Paulo, São Carlos, 13560-924, Brazil, [2]Department of Computer Science, Federal University of Technology-Paraná (UTFPR), Cornélio Procópio, 80230-901, Brazil and [3]Global South Artificial Intelligence for Pandemic and Epidemic Preparedness & Response Network (AI4PEP)

*Corresponding author. bonidia@utfpr.edu.br, andre@icmc.usp.br

FOR PUBLISHER ONLY Received on Date Month Year; revised on Date Month Year; accepted on Date Month Year

Abstract

Supplementary Files

Selected Studies: Summary, Strengths, and Gaps

Regarding automation, the tools were divided into three groups: Web servers, those that claim to be end-to-end, and those that do not. End-to-end tools are those capable of automatically performing all stages to develop a predictive model, from descriptor extraction to tuning the hyperparameters of the final model. As for documentation, three levels were evaluated: documentation for new experiments (new exp.), which teaches how to apply the tool to new datasets; reproducibility documentation (reprod.), which instructs how to create a model with the data reported in the paper; and undocumented tools, which do not provide any guidance for executing the code on datasets. This information is summarized in Figure 1.

First, there are 13 web servers, where, in most cases, the user can predict new interactions based on a pre-trained model. These servers typically have user-friendly interfaces and documentation that guide their use. Next, the search returned six end-to-end tools with complete documentation, which means these tools perform the entire model development pipeline and provide detailed guidance to facilitate their use in new experiments. Additionally, two end-to-end tools were identified, which, although capable of building a model from scratch, do not provide documentation, making it difficult to replicate and adapt their code.

As for the non-end-to-end tools, 20 tools that have documentation teaching how to apply them to new data were identified, allowing some flexibility in adapting the methods. In addition to these, 15 non-end-to-end tools have documentation restricted to application on the data from the article itself, focusing on reproducibility. Finally, 30 non-end-to-end tools were classified as undocumented, as they do not provide instructions for executing or replicating their code on any dataset.

This categorization, shown in Figure 1, highlights the diversity of available studies, both in terms of functionality and user support. The group of tools that are the most accessible is the web servers, as they do not require any interaction with programming. However, most of these servers have the limitation of operating with pre-trained predictive models in specific contexts, restricting their applicability to predefined scenarios.

On the other hand, end-to-end tools provide a more comprehensive approach, allowing the training of custom models for specific data. Among them, tools with complete documentation are the most recommended as they guide the user through the entire process, from feature descriptor extraction to model tuning.

These two groups, web servers and well-documented end-to-end tools, represent only a quarter of all the tools considered in this mapping. This already provides an initial indication that tools that require low knowledge of programming and ML make up a small fraction of the studies analyzed. Most tools require some level of technical involvement, which limits their use for users without prior knowledge in the development of predictive models.

Web Servers and End-to-End Tools Bibliometrics

Table 1 presents a compilation of tools that offer web servers and their main characteristics. The table includes the tool's name, the year of release, the type of interaction, whether it only predicts interactions based on a pre-trained model or can train a model with user-supplied data, the available model organisms that are fed to the pre-trained models or that, according to the authors, can be predicted, and finally, the status of whether it is still active or not (verified on february 19, 2026). Note that all listed tools use only the primary structure of biological sequences as input. On the other hand, the Table 2 summarizes the characteristics of end-to-end tools.

© The Author 2022. Published by Oxford University Press. All rights reserved. For permissions, please e-mail: journals.permissions@oup.com

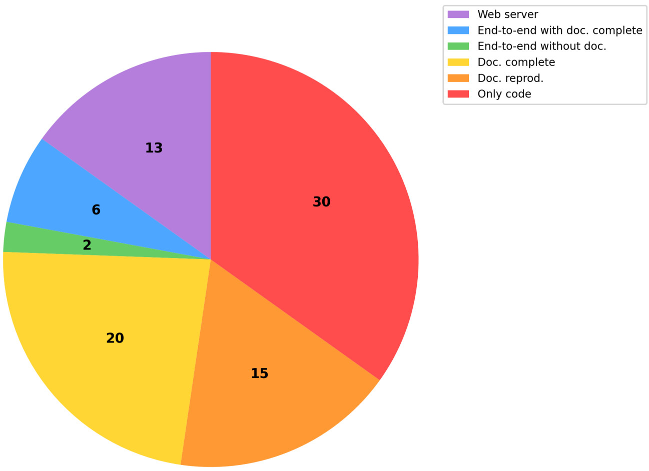

Fig. 1. First division regarding automation and documentation of studies for interaction prediction.

Table 1. Table presenting the web servers identified in the review.

Tool	Year	Task	Fitting or apply a pre-trained model	Organism / group model	Status (Active/Inactive)
BeRBP [1]	2019	RPI	only pre-trained	human	Inactive
InterSPPI [2]	2020	PPI	only pre-trained	human-virus, human-bacteria, arabidopsis thaliana-pathogens	Active
Bot-Net [3]	2021	RRI	fit and apply a pre-trained	human	Inactive
LSTM-PHV [4]	2021	PPI	only pre-trained	human-virus	Active
HVIDB [5]	2021	PPI	only pre-trained	human-virus	Active
ProteinPrompt [6]	2022	PPI	only pre-trained	human, mamalia, vertebrates, metazoa	Active
deepHPI [7]	2022	PPI	only pre-trained	human-bacteria, human-virus, plant-pathogen, animal-pathogen	Inactive
DeepPPAPred [8]	2023	PPI	only pre-trained	human-pathogen	Active
DeepAraPPI [9]	2023	PPI	only pre-trained	arabidopsis thaliana, oryza sativa	Active
INTREPPPID [10]	2024	PPI	only pre-trained	human, mouse, zebrafish, fruit fly, nematode, thales cress	Inactive
LncPTPred [11]	2025	RPI	only pre-treined	human	Active
PlantPathoPPI [12]	2025	PPI	only pre-treined	plant-pathogen	Active
AttnSeq-PPI [13]	2026	PPI	only pre-treined	human	Active

Analysis of Annual Trends: Quantity, Accessibility and Validation

This section discusses trend analyses related to the selected studies identified by the authors as relevant. It starts by presenting the distribution of tools designed to predict PPIs, RPIs, and RRIs, as reported in Figure 2. An important note is that some studies found worked with more than one category of interaction, thus contributing to more than one problem.

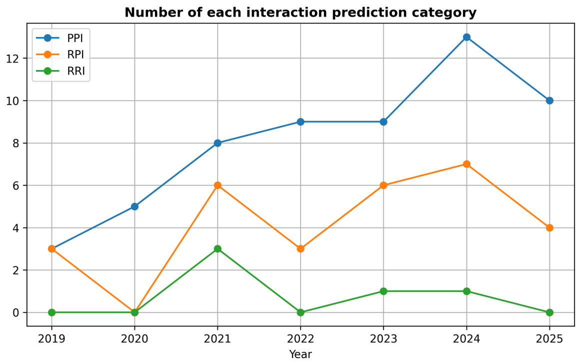

Fig. 2. Number of tools for each category of biological sequence interaction per year.

A significant increase is observed in the trend of studies on interaction prediction from 2020 to 2021 across the three categories of prediction analyzed. In particular, the PPI tools showed a constant increase until 2022, followed by a stable year and another significant increase in 2024. In contrast, the RPI and RRI tools exhibit a more irregular pattern, with peaks in 2021 and 2023. Initially, we observed rapid growth in this area in recent years, particularly in the PPI domain.

The growth in this area can be attributed to two main factors. The first factor is the release of AlphaFold in 2020, which revolutionized the prediction of the tertiary structure of the protein [21]. Although this information is not directly used by many studies, possibly because of high computational costs, it enabled further analyses and boosted computational studies of proteins.

This is the case of the STRING PPI database, which allows the visualization of the structures of proteins involved in interactions through AlphaFold, providing additional information to users [22]. The second factor was the COVID-19 pandemic, also in 2020, which stimulated investments in molecular biology, especially in techniques capable of accelerating research on biological processes and the development of new approaches, involving both proteins and RNAs, as some vaccines produced are based on RNA-protein interactions [23].

In addition, Figure 3 shows the percentage of end-to-end applications, web servers, and non-end-to-end tools per year. It is noted that most tools are non-end-to-end, meaning they were

Table 2. End-to-end tools for predicting interactions between biological sequences. The table records the year of publication of the study, the type of predictive task, the level of documentation, the type of input, the classification of the predictive model, the existence of outputs related to interpretability (Interpr.) and user decision-making (Dec. Mak.), and finally, the number of datasets and tools compared during validation.

Tool	Year	Task	Documentation	Input	Model type	Interpr.	Dec. Mak.	Nº datasets	Nº tools
Rpi-mdlstack [14]	2022	RPI	No doc.	1º struc.	White and Black box	-	-	6	7
HDRNet [15]	2023	RPI	New exp.	1º struc. and icShape	Black box	-	X	261	6
ncrpi-lgat [16]	2023	RPI	New exp.	1º struc.	Black box	-	-	3	4
TAGPPI [17]	2022	PPI	No doc.	1º struc.	Black box	-	-	8	8
Struct2graph [18]	2022	PPI	New exp.	1º and 3º struc.	Black box	-	X	5	8
HIGH-PPI [19]	2023	PPI	New exp.	1º and 3º struc.	Black box	X	X	6	6
INTREPPPID [10]	2024	PPI	New exp.	1º struc.	Black box	-	X	4	5
BioPrediction-RPI [20]	2024	RPI	New exp.	1º struc.	White box	X	-	12	12

not designed to be fully automated and capable of handling different datasets independently.

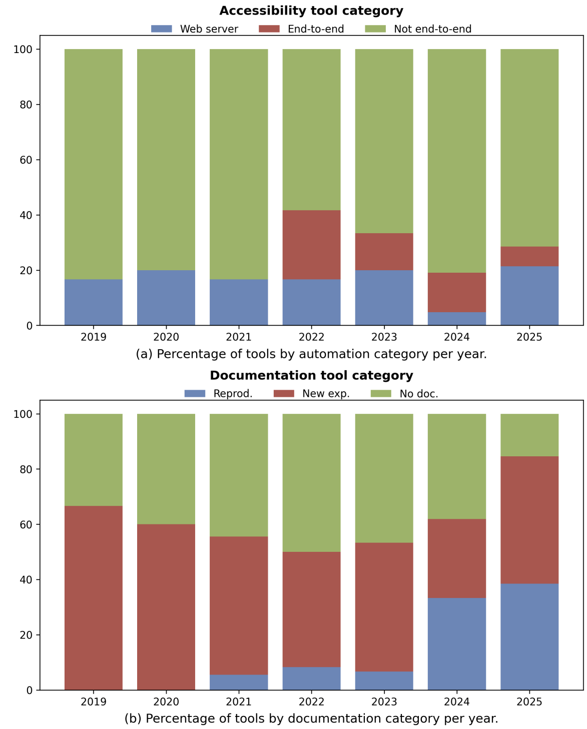

(a) Percentage of tools by automation category per year.

(b) Percentage of tools by documentation category per year.

Fig. 3. Annual trends of tools by category and regarding accessibility for non-specialists.

However, starting in 2022, the first tools that claim to be end-to-end emerged, one year after the significant increase in the area of interaction predictions, as seen in Figure 3a. This suggests that with the increase in studies in the area, new approaches have been developed to address the problem, or that the techniques have become relatively robust to be integrated into frameworks capable of automatically handling diverse scenarios.

In addition, the percentage of web servers made available has remained relatively constant over the years. Most of the models available are pre-trained to make predictions in specific scenarios, which may explain the lack of growth in this approach. Unfortunately, many web servers are designed to work only in a specific context, limiting their appeal and applicability in broader scenarios. Therefore, it is often more effective for a researcher to fine-tune a model for their particular case, ensuring specificity.

Figure 3b presents the temporal analysis of the documentation category available for the tools. It is observed that, although the number of tools with some documentation category has remained relatively constant, there is an increase in the predominance of documentation that only reproduces the data from the original article, as opposed to more comprehensive documentation that guides the application of the tools to new data.

The authors also constructed Figure 4a, which shows the number of tools per year that include outputs related to interpretability or that aim to assist the user in applying the model (decision-making report).

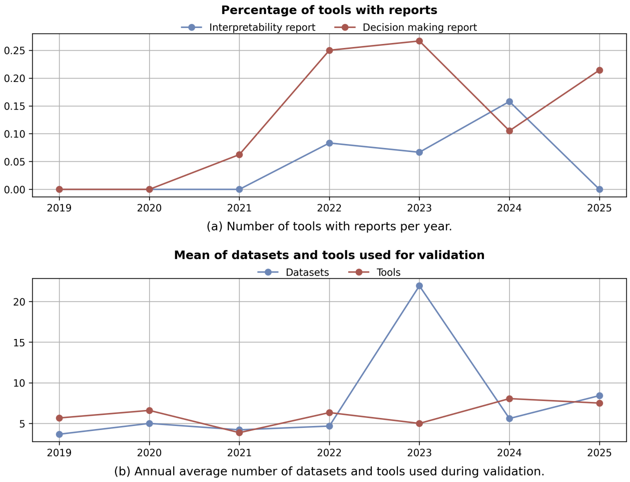

(a) Number of tools with reports per year.

(b) Annual average number of datasets and tools used during validation.

Fig. 4. Annual trends of tools regarding complementary outputs and validation process extent.

The authors observed that, in 2019 and 2020, no tool included the generation of decision-making and interpretability reports. Only from 2022 onward were studies with these characteristics found, the same year in which the first end-to-end tools appeared. This may indicate that the same movement that led to the development of automated tools also influenced the creation of tools capable of providing more information to the user than just the prediction itself. Even in the years with the highest number of recorded reports, only about 23% of the tools included outputs related to interpretability and 25% to model decision-making. In other words, there is a growing trend toward developing more comprehensive outputs for users, but this practice is still adopted by a minority of the tools.

Next, Figure 4b is presented, which highlights the average number of datasets and benchmarked tools used during the

validation process over the years. It is noticeable that in 2023, the average number of datasets employed during validation shows a significant disparity compared to previous years. This disparity is attributed to an outlier study that stands out for using 261 datasets during the validation phase, HDRNet [15], which consequently resulted in a substantial increase in the average for that specific period.

When excluding this outlier study, the average number of datasets drops to 4.93 and 8.42 in the two consecutive years, a value consistent with those observed in other years. This analysis reveals that, when disregarding the mentioned study, the annual average of datasets used during validation, as well as the number of other tools employed for performance comparison, remains relatively stable. This stability highlights the consistency of the standard practices adopted in the state-of-the-art for conducting the validation process.

Analysis of Annual Trends in Modeling: Inputs, Feature Descriptor, and Modeling Strategies

This section presents a set of graphs illustrating the frequency of additional descriptors observed in each selected study, as reported in Figure 5. These descriptors are organized into three categories: the category of input provided to the model, the category of feature descriptor extracted from the input, and the category of modeling strategy employed.

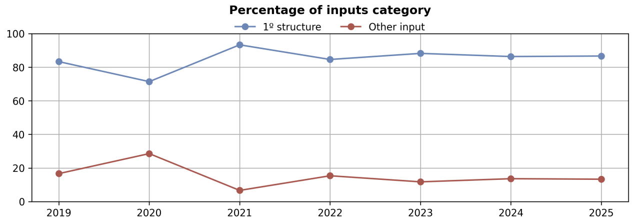

(a) Frequency of each input category per year.

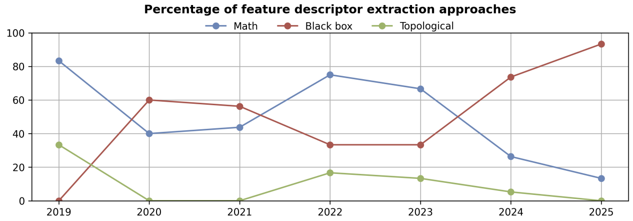

(b) Frequency of each feature descriptor category per year.

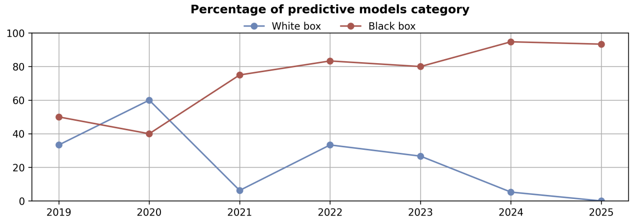

(c) Frequency of each modeling strategy used per year.

Fig. 5. Annual trends of the tools regarding input, feature descriptor, and modeling strategy.

This classification provides an overview of the approaches adopted by each study, from the input stage to the modeling stage. However, this will not be the main focus of the mapping as other secondary studies explore this topic in more depth. Thereby, Figure 5a presents the proportions of each category of input used to characterize each molecule (in addition to known interactions used for model training) over the years, divided into two categories.

The first column refers to the use of the primary structure of each sequence (1º structure), while the second includes less common input types (Other type), such as GO, tertiary structure, experimental data, and SMILES annotations of the sequences. It is observed that the primary structure remains the main input used by the tools over the years. However, in some cases, these tools also make complementary use of tertiary structure and other data sources, such as GO and in vivo experimental information, aiming to enrich the models and improve prediction accuracy.

Figure 5b presents a column that classifies the type of feature descriptor used by each tool into three categories: mathematical, which extracts patterns from the structure of each sequence using mathematical techniques; black box, which uses embeddings based on DL or Large Language Models (LLMs) for feature descriptor extraction; and topological, which captures information directly from known interaction networks.

The authors observed that embedding-based techniques began to be widely adopted in 2020. However, they have not become dominant in the state-of-the-art, as mathematical techniques remain extensively employed. In parallel, topological features descriptors, derived from known interaction networks, represent a less common approach, yet they are still occasionally used to enrich the decision-making process.

Finally, Figure 5c presents the category of modeling strategy used for training, classified into two groups: the white box, which includes traditional ML models, and the black box, which refers to DL-based approaches. Note that in some cases, models combine techniques from different categories, such as the integration of white-box and black-box models. It can be observed that, in most years, tools have predominantly relied on black-box models. In addition, there is a noticeable decline in the use of traditional models (white-box). In contrast, the use of black-box models continues to grow, approaching near-total dominance in the field.

Classical Approaches Description

In parallel, the prediction of biomolecular interactions represents a well-established research field, with numerous studies developed long before the emergence of ML models. To contextualize these traditional approaches, we present below a representative selection, highlighting their main characteristics, the principles underlying their predictions, and their modes of use.

STRING [24, 25] is a widely used database for predicting functional associations between proteins, which include, but are not limited to, direct physical interactions. Its predictions are obtained through the integration of multiple independent sources of evidence, primarily grounded in comparative genomics, classical statistical methods, and curated biological knowledge. These evidence types include conserved gene neighborhood, gene fusion, phylogenetic co-occurrence, gene co-expression, experimentally derived interactions, curated pathway and protein complex databases, and text mining of the scientific literature. Each evidence source produces an independent probabilistic score, which is subsequently normalized and combined into a global confidence score.

This score reflects the likelihood of a biologically meaningful functional association rather than exclusively a physical protein–protein interaction, a conceptual hallmark of the STRING methodology. [24]. Currently, STRING comprises approximately 20 billion protein–protein associations involving around 60 million proteins across more than 10,000 organisms. Although it does not allow users to generate novel predictions directly, since it is a repository of precomputed interactions, the platform enables querying interactions by protein or species, as well as the visualization of interaction networks and the biological functions of proteins within these networks.

ClusPro [26] is a web server for the structural prediction of protein–protein interactions through molecular docking. The protocol requires as input two protein structures in PDB format and is based on global sampling of conformational space, followed by evaluation using physics-based statistical energy functions. The generated solutions are clustered according to structural similarity, and the centers of the most populated low-energy clusters are selected as the final models. The server also provides additional options, such as the application of docking restraints and the removal of intrinsically disordered regions, while maintaining computational efficiency compatible with large-scale analyses.

HDOCK [27] is a web server for the structural prediction of PPIs, RPIs, and protein–DNA interactions (PDIs) that employs a hybrid strategy combining template-based modeling with free (ab initio) docking. The method exploits structural information from the Protein Data Bank (PDB) when suitable templates are available, but resorts to ab initio docking when templates are absent or unreliable. HDOCK accepts either sequences or structures as input, is computationally efficient (10–20 minutes per run), and demonstrates robust performance even for complexes with low sequence identity, being particularly effective in the prediction of RPIs.

NPDock [28] is a web server designed for the structural prediction of RPIs and PDIs through computational docking based on individual molecular structures. The method implements a workflow that includes the generation of docking poses, scoring using dedicated energy functions, clustering of the top-ranked solutions, and structural refinement of the most promising models. As minimal input, the server requires protein and nucleic acid structures in PDB format, and it also provides advanced options to control the docking process as well as three-dimensional visualization of the predicted complexes.

RBPmap [29] is a web server dedicated to the prediction and mapping of binding sites of RNA-binding proteins (RBPs) on both coding and non-coding RNAs. The tool is based on the identification of experimentally defined binding motifs, provided either from a curated database or by the user, represented as consensus sequences or position-specific scoring matrices (PSSMs). The algorithm employs a weighted ranking approach that accounts for binding-site clustering tendencies and the evolutionary conservation of regulatory regions. In addition, it uses context-specific background models for different genomic regions, such as untranslated regions (UTRs), splicing sites, and non-coding RNAs, enabling accurate identification of potential RPI-related binding regions.

RNAplex [30, 31] is a program designed for the fast prediction of RRIs, with a particular focus on identifying hybridization sites of regulatory RNAs in large-scale datasets. The tool employs a simplified energy model that substantially reduces computational cost compared to classical RNA cofolding approaches, while still retaining the ability to detect energetically stable interactions. In addition, RNAplex incorporates a length penalty, allowing the prioritization of short and highly stable interactions. This feature makes it especially suitable for large-scale analyses of non-coding RNAs, including ncRNAs, miRNAs, and siRNAs.

IntaRNA [32, 31] is a computational tool developed for the prediction of RRIs, with particular emphasis on identifying bacterial sRNA targets. The method explicitly incorporates the accessibility of interaction sites on both RNA molecules, as well as the presence of a user-defined seed region for base pairing, thereby overcoming limitations of approaches that neglect these factors or lack scalability for genome-wide searches. Benchmark evaluations indicate that IntaRNA achieves improved precision in identifying exact RNA–RNA interaction sites while maintaining computational efficiency suitable for large-scale analyses.

RNAup [33, 31] is a tool dedicated to the thermodynamic prediction of RRIs, based on decomposing the binding free energy into two components: the energy required to make the target site accessible and the hybridization energy between the interacting RNAs. The method extends the classical partition function approach for RNA secondary structures, enabling the calculation of the probability that specific regions are unpaired and thus available for interaction. This modeling framework allows a rigorous estimation of binding free energies between small regulatory RNAs, such as siRNAs, and large mRNA targets, showing good correlation with experimental data while maintaining a computational cost suitable for large-scale analyses.

In general, traditional techniques for predicting biomolecular interactions, such as physical docking (e.g., ClusPro [26], HDOCK [27], NPDock [28]), motif- and domain-based methods (e.g., RBPmap [29]), and thermodynamic models for RRIs (e.g., RNAplex [30], IntaRNA [32], RNAup [33]), are grounded in well, established physicochemical, evolutionary, and statistical principles. These approaches offer high interpretability, a strong mechanistic connection to the underlying biological phenomena, and robust performance in data-scarce scenarios. Moreover, they are widely accepted as methodological references in experimental studies and are broadly available to the scientific community, predominantly through web-based servers.

References

1. Hui Yu, Jing Wang, Quanhu Sheng, Qi Liu, and Yu Shyr. beRBP: binding estimation for human RNA-binding proteins. *Nucleic Acids Research*, 47(5):e26, March 2019. Research Support, N.I.H., Extramural.
2. Xiaodi Yang, Shiping Yang, Qinmengge Li, Stefan Wuchty, and Ziding Zhang. Prediction of human-virus protein-protein interactions through a sequence embedding-based machine learning method. *Computational and Structural Biotechnology Journal*, 18:153–161, December 2020. © 2019 The Authors.
3. Muhammad N. Asim, Muhammad A. Ibrahim, Cornelia Zehe, and et al. BoT-Net: a lightweight bag of tricks-based neural network for efficient LncRNA–miRNA interaction prediction. *Interdisciplinary Sciences: Computational Life Sciences*, 14:841–862, 2022.
4. Sho Tsukiyama, Md Mehedi Hasan, Satoshi Fujii, and Hiroyuki Kurata. LSTM-PHV: prediction of human-virus protein–protein interactions by LSTM with word2vec. *Briefings in Bioinformatics*, 22(6), November 2021.

5. Xiaodi Yang, Xiaomin Lian, Chen Fu, Stefan Wuchty, Shiping Yang, and Ziding Zhang. HVIDB: a comprehensive database for human-virus protein-protein interactions. *Briefings in Bioinformatics*, 22(2):832–844, March 2021.
6. Sebastian Canzler, Markus Fischer, Daniel Ulbricht, Nenad Ristic, Peter W. Hildebrand, and Ralph Staritzbichler. ProteinPrompt: a webserver for predicting protein-protein interactions. *Bioinform Adv*, 2(1):vbac059, August 2022.
7. Rakesh Kaundal, Camilo D. Loaiza, Nitish Duhan, and Nathaniel Flann. deepHPI: a comprehensive deep learning platform for accurate prediction and visualization of host-pathogen protein-protein interactions. *Briefings in Bioinformatics*, 23(3):bbac125, May 2022.
8. Rahul Nikam, Kanchan Yugandhar, and Michael M. Gromiha. Deep learning-based method for predicting and classifying the binding affinity of protein-protein complexes. *Biochimica et Biophysica Acta (BBA) - Proteins and Proteomics*, 1871(6):140948, November 2023.
9. Jingyan Zheng, Xiaodi Yang, Yan Huang, Shiping Yang, Stefan Wuchty, and Ziding Zhang. Deep learning-assisted prediction of protein-protein interactions in arabidopsis thaliana. *The Plant Journal*, 114(4):984–994, May 2023. Epub 2023 Mar 29.
10. J. Szymborski and A. Emad. Intrepppid-an orthologue-informed quintuplet network for cross-species prediction of protein-protein interaction. *Briefings in Bioinformatics*, 25(5):bbae405, 2024.
11. Gourab Das, Troyee Das, and Zhumur Ghosh. Lncptpred: predicting lncrna–protein interaction based on crosslinking and immunoprecipitation (clip-seq) data. *Briefings in Bioinformatics*, 26(4):bbaf432, 08 2025.
12. Sneha Murmu, Himanshushekhar Chaurasia, A.R. Rao, Anil Rai, Sarika Jaiswal, Anshu Bharadwaj, Rajbir Yadav, and Sunil Archak. Plantpathoppi: An ensemble-based machine learning architecture for prediction of protein-protein interactions between plants and pathogens. *Journal of Molecular Biology*, 437(15):169093, 2025. Computation Resources for Molecular Biology.
13. Dipayan Sarkar and Chiranjib Sarkar. Attnseq-ppi: Enhancing protein-protein interaction network prediction using transfer learning-driven hybrid attention. *Biochimica et Biophysica Acta (BBA) - Proteins and Proteomics*, 1874(1):141102, 2026.
14. Bin Yu, Xue Wang, Yaqun Zhang, Hongli Gao, Yifei Wang, Yushuang Liu, and Xin Gao. Rpi-mdlstack: Predicting rna–protein interactions through deep learning with stacking strategy and lasso. *Applied Soft Computing*, 120:108676, 2022.
15. Hao Zhu, Yu Yang, Yihong Wang, and et al. Dynamic characterization and interpretation for protein-RNA interactions across diverse cellular conditions using HDRNet. *Nature Communications*, 14:6824, 2023.
16. Yong Han and Shao-Wu Zhang. ncrpi-lgat: Prediction of ncrna-protein interactions with line graph attention network framework. *Computational and Structural Biotechnology Journal*, 21:2286–2295, 2023.
17. B. Song, X. Luo, X. Luo, Y. Liu, Z. Niu, and X. Zeng. Learning spatial structures of proteins improves protein-protein interaction prediction. *Brief Bioinform*, 23(2):bbab558, Mar 2022.
18. Mayank Baranwal, Alec Magner, Joseph Saldinger, et al. Struct2graph: a graph attention network for structure based predictions of protein–protein interactions. *BMC Bioinformatics*, 23:370, 2022.
19. Zhen Gao, Cheng Jiang, Jun Zhang, et al. Hierarchical graph learning for protein–protein interaction. *Nature Communications*, 14:1093, 2023.
20. Bruno Rafael Florentino, Robson Parmezan Bonidia, Natan Henrique Sanches, Ulisses N. da Rocha, and André C.P.L.F. de Carvalho. Bioprediction-rpi: Democratizing the prediction of interaction between non-coding rna and protein with end-to-end machine learning. *Computational and Structural Biotechnology Journal*, 23:2267–2276, 2024.
21. Oleg Kovalevskiy, Juan Mateos-Garcia, and Kathryn Tunyasuvunakool. Alphafold two years on: Validation and impact. *Proceedings of the National Academy of Sciences*, 121(34):e2315002121, 2024.
22. Mihaly Varadi and Sameer Velankar. The impact of alphafold protein structure database on the fields of life sciences. *PROTEOMICS*, 23(17):2200128, 2023.
23. Koen Bruynseels. Responsible innovation in synthetic biology in response to covid-19: the role of data positionality. *Ethics and Information Technology*, 23(Suppl 1):117–125, 2021.
24. Christian von Mering, Lars J. Jensen, Berend Snel, Sean D. Hooper, Markus Krupp, Mathilde Foglierini, Nelly Jouffre, Martijn A. Huynen, and Peer Bork. String: known and predicted protein–protein associations, integrated and transferred across organisms. *Nucleic Acids Research*, 33(Database issue):D433–D437, January 2005.
25. Damian Szklarczyk, Katerina Nastou, Maria Koutrouli, Ronny Kirsch, Farrokh Mehryary, Reza Hachilif, Di Hu, Michael E. Peluso, Qiang Huang, Tao Fang, Nadezhda T. Doncheva, Sampo Pyysalo, Peer Bork, Lars J. Jensen, and Christian von Mering. The string database in 2025: protein networks with directionality of regulation. *Nucleic Acids Research*, 53(D1):D730–D737, January 2025.
26. Dima Kozakov, David R. Hall, Bing Xia, Kathryn A. Porter, Dzmitry Padhorny, Christine Yueh, Dmitri Beglov, and Sandor Vajda. The cluspro web server for protein–protein docking. *Nature Protocols*, 12(2):255–278, 2017.
27. Yao Yan, Dapeng Zhang, Pei Zhou, Bin Li, and Sheng-You Huang. Hdock: a web server for protein-protein and protein-dna/rna docking based on a hybrid strategy. *Nucleic Acids Research*, 45(W1):W365–W373, 2017.
28. Irina Tuszynska, Marcin Magnus, Katarzyna Jonak, Wayne Dawson, and Janusz M. Bujnicki. Npdock: a web server for protein–nucleic acid docking. *Nucleic Acids Research*, 43(W1):W425–W430, 2015.
29. Itay Paz, Irit Kosti, Manuel Jr. Ares, Melissa Cline, and Yael Mandel-Gutfreund. Rbpmap: a web server for mapping binding sites of rna-binding proteins. *Nucleic Acids Research*, 42(W1):W361–W367, 2014.
30. Hakim Tafer and Ivo L. Hofacker. Rnaplex: a fast tool for rna–rna interaction search. *Bioinformatics*, 24(22):2657–2663, 04 2008.
31. Sebastian U. Umu and Paul P. Gardner. A comprehensive benchmark of rna-rna interaction prediction tools for all domains of life. *Bioinformatics*, 33(7):988–996, 2017.
32. Anke Busch, Andreas S. Richter, and Rolf Backofen. Intarna: efficient prediction of bacterial srna targets incorporating target site accessibility and seed regions. *Bioinformatics*, 24(24):2849–2856, 10 2008.
33. Ulrike Mückstein, Hakim Tafer, Stephan H. Bernhart, Maribel Hernandez-Rosales, Jörg Vogel, Peter F. Stadler, and Ivo L. Hofacker. Translational control by rna-rna interaction: Improved computation of rna-rna binding

thermodynamics. In Mourad Elloumi, Josef Küng, Michal Linial, Robert F. Murphy, Kristan Schneider, and Cristian Toma, editors, *Bioinformatics Research and Development*, pages 114–127, Berlin, Heidelberg, 2008. Springer Berlin Heidelberg.
